# Is acupuncture a useful adjunct to physiotherapy for older adults with knee pain?: The "Acupuncture, Physiotherapy and Exercise" (APEX) study [ISRCTN88597683]

**DOI:** 10.1186/1471-2474-5-31

**Published:** 2004-09-02

**Authors:** Elaine Hay, Panos Barlas, Nadine Foster, Jonathan Hill, Elaine Thomas, Julie Young

**Affiliations:** 1 Primary Care Sciences Research Centre, Keele University, Keele, North Staffordshire, United Kingdom, ST5 5BG; 2 Staffordshire Rheumatology Centre, The Haywood, Burslem, Stoke-on-Trent, North Staffordshire, United Kingdom, ST6 7AG; 3 School of Health & Rehabilitation, Keele University, Keele, North Staffordshire, United Kingdom, ST5 5BG

## Abstract

**Background:**

Acupuncture is a popular non-pharmacological modality for treating musculoskeletal pain. Physiotherapists are one of the largest groups of acupuncture providers within the NHS, and they commonly use it alongside advice and exercise. Conclusive evidence of acupuncture's clinical effectiveness and its superiority over sham interventions is lacking. The Arthritis Research Campaign (**arc**) has funded this randomised sham-controlled trial which addresses three important questions. Firstly, we will determine the additional benefit of true acupuncture when used by physiotherapists alongside advice and exercise for older people presenting to primary care with knee pain. Secondly, we will evaluate sham acupuncture in the same way. Thirdly, we will investigate the treatment preferences and expectations of both the participants and physiotherapists participating in the study, and explore the effect of these on clinical outcome. We will thus investigate whether acupuncture is a useful adjunct to advice and exercise for treating knee pain and gain insight into whether this effect is due to specific needling properties.

**Methods/Design:**

This randomised clinical trial will recruit 350 participants with knee pain to three intervention arms. It is based in 43 community physiotherapy departments in 21 NHS Trusts in the West Midlands and Cheshire regions in England. Patients aged 50 years and over with knee pain will be recruited. Outcome data will be collected by self-complete questionnaires before randomisation, and 6 weeks, 6 months and 12 months after randomisation and by telephone interview 2 weeks after treatment commences. The questionnaires collect demographic details as well as information on knee-related pain, movement and function, pain intensity and affect, main functional problem, illness perceptions, self-efficacy, treatment preference and expectations, general health and quality of life. Participants are randomised to receive a package of advice and exercise; or this package plus real acupuncture; or this package plus sham acupuncture. Treatment details are being collected on a standard proforma. Interventions are delivered by experienced physiotherapists who have all received training in acupuncture to recognised national standards. The primary analysis will investigate the main treatment effects of real or sham acupuncture as an adjunct to advice and exercise.

**Discussion:**

This paper presents detail on the rationale, design, methods, and operational aspects of the trial.

## Background

Knee pain in older adults is a common disabling problem. Approximately 25% of the population aged over 55 years are affected at any one time and half of these will have some restriction of normal daily activities [1,2]. After excluding `red flags' and specific pathologies such as inflammatory arthritis, most knee pain in older adults is due to osteoarthritis. Controlling the pain and minimising loss of function are the principal aims of treatment. Most sufferers are managed exclusively in primary care [3-5], where the usual approaches include analgesics and exercise [6-11]. A report from Arthritis Care [12] of patients' perspectives highlighted that people with knee osteoarthritis want treatment offering more pain relief and help with mobility. Easy to understand information was also felt to be important, as was exercise, to help manage the problem. A recent review of international guidelines suggests that, for patients with knee pain, the best non-pharmacological care consists of education, muscle strengthening and exercise [13].

Patients with musculoskeletal pain often choose methods of treatment that are not widely available within the NHS, such as complementary medicine [14]. Reports from the United States and the United Kingdom have indicated the popularity of complementary medicine with the general public and health care professionals [15-19]. Complementary medicine is available in approximately 40% of general practice surgeries and general practitioners and physiotherapists are the largest providers of complementary medicine within the NHS [17]. Acupuncture is one of the most popular complementary medicine modalities in the UK: reports suggest that it is available in 84% of chronic pain clinics and approximately 4000 general practitioners and physiotherapists are trained in acupuncture [21,22]. Although recent authors have promoted the concept of integrated practice incorporating conventional and complementary therapies [20], current guidelines highlight the need for further research evidence for the use of acupuncture for knee pain in older adults [13]. The clinical effectiveness of acupuncture, and the question of whether it is superior to sham interventions has not been established. In addition to providing exercise and advice, physiotherapists are also one of the largest groups of acupuncture providers within both primary and secondary care in the NHS [23]. Physiotherapy is therefore an appropriate and important arena in which to investigate the effectiveness of integrated mainstream and complementary therapy.

## Evidence for advice and exercise

International guidelines suggest that the best package of care for this patient group is one that includes patient education, advice and exercise [13]. There is strong evidence for the usefulness of education, muscle strengthening and aerobic exercise. The beneficial effects of exercise on knee pain are well documented and it is a key component of successful rehabilitation programmes for patients [24,25]. Active rehabilitation programmes for patients with musculoskeletal and arthritic pain not only improve joint function and reduce pain, but also improve strength, walking speed and self-efficacy [26] as well as quality of life, and they reduce risk of other chronic conditions [27]. Randomised clinical trials consistently show the benefit of exercise for knee pain in older adults [28-31]. Recent studies also highlight the need to provide adequate instruction, feedback and practice in order to ensure that the key muscle groups around the knee, such as the quadriceps, are activated [32]. The European League Against Rheumatism (EULAR) recommendations have recently been updated and in particular, advocate exercise for knee pain related to osteoarthritis [33]. In line with this evidence base, the current trial was designed so that all participants receive a package of care which includes education, advice, and exercise.

## Evidence for acupuncture

The physiological properties of acupuncture have been well described in the laboratory. Acupuncture activates central mechanisms of pain control and elicits release of specific neurotransmitters (mainly opioids) in the central nervous system [34,35]. Effects on the autonomic nervous system have also been demonstrated during and after acupuncture stimulation [36,37]. Despite this, its clinical effectiveness remains a matter of controversy [38,39]. This is partly because of methodological limitations in many trials of acupuncture, including small sample sizes, lack of credible sham-controls, and inadequate blinding [40]. Acupuncture has been shown to have a short-term analgesic effect in musculoskeletal pain [41,42]. A recent evaluation of acupuncture by the National Institutes of Health concluded that it has an analgesic effect on dental and orofacial pain and is a useful adjunct in a range of painful conditions, including musculoskeletal and myofascial pain [43]. In fibromyalgia, there is increasing evidence demonstrating the usefulness of acupuncture [44,45]. One meta-analysis concluded that acupuncture might offer benefit to patients with knee osteoarthritis when used as an adjunct to mainstream management strategies [46].

Appropriate sham interventions for acupuncture have been widely debated and several placebo needles have been introduced and tested [47,48]. A trial conducted in Germany recently concluded that true acupuncture has a better effect than sham acupuncture in the treatment of knee and back pain, but not for migraine headache [49]. In addition, another study reported positive effects of acupuncture for knee pain [50]. However, a key limitation to these studies is the lack of long-term follow-up, something which the current study has been designed to address [51].

We have designed, and are currently implementing, a prospective sham controlled randomised trial within the primary care setting addressing the important clinical question: is acupuncture a useful adjunct to physiotherapy care (advice and exercise) for treating knee pain in older adults? Research and development in primary care is important to public health and necessary to support the decisions and treatments in this setting [52].

The primary objective is to compare, at 6 months, the clinical outcomes of true acupuncture plus advice and exercise, with advice and exercise alone for treating people aged 50 years and over referred directly from primary care with knee pain. Our secondary objectives are

i) to compare, at 6 weeks and 12 months, the clinical outcomes of adding true acupuncture to advice and exercise alone, in the same patient group.

ii) to compare, at 6 weeks, 6 and 12 months, the clinical outcomes of sham acupuncture plus advice and exercise, with advice and exercise alone, in the same patient group.

iii) to measure patients and physiotherapists beliefs, preferences and expectations about the treatments being tested and to explore their effect on clinical outcome.

## Methods

### Trial design

This multicentre, three-arm sham-controlled randomised trial will be conducted in 43 individual Physiotherapy Centres that provide services for Primary Care Physicians located in 21 NHS Trusts situated in the Midlands and Cheshire regions of the UK. Multi-centre ethical approval has been obtained from the West Midlands Multicentre Research Ethics Committee and local approval was given by 12 ethics committees (Southern Derbyshire, Shropshire, Worcestershire, Warwickshire, Mid Staffordshire, South Staffordshire, Sollihull, North Birmingham, South Birmingham, West Birmingham, East Birmingham, East Cheshire). The trial was designed by a steering group with expert input from physiotherapists, an acupuncture specialist and trial methodologists. Information will be collected from the individual participating physiotherapists (demographics, current training and use of acupuncture, attitudes and beliefs about knee pain, and beliefs and expectations of the three treatment packages being compared in the trial) prior to the commencement of the trial and after the trial has been completed (Table 1).

### Study population

Participants include patients with knee pain aged 50 years and over referred to physiotherapy centres by their general practitioner. Participants will be randomised to one of three groups: (i) advice and exercise alone, (ii) advice and exercise plus true acupuncture, (iii) advice and exercise plus sham acupuncture. Follow up will be at 2 weeks (by telephone), 6 weeks, 6 months and 12 months after randomisation, by postal questionnaire. Non-responders will be followed up.

### Inclusion criteria

Eligible patients are male and female subjects aged 50 years and above with pain (with or without stiffness) in one or both knees presenting to primary care. They must be naïve to acupuncture treatment (i.e. have never experienced acupuncture before for their present or any past complaints), and considered suitable for referral to a physiotherapy outpatients department by their general practitioner. Participants must be able to read and write English, be willing to consent to participation, and able give full informed consent. They must also be available for telephone contact.

### Exclusion criteria

Patients with potentially serious pathology (e.g. inflammatory arthritis, malignancy etc) on the basis of general practice or physiotherapy diagnosis or from past medical history, those who have had a knee or hip replacement on the affected side(s), are already on a surgical waiting list for total knee replacement, or for whom the trial interventions are contraindicated are excluded from the trial. Those who have received an exercise programme, from a physiotherapist, for their knee problem within the last 3 months (normal recreational involvement in sport or exercise will not be an exclusion) or an intra-articular injection to the knee in the last 6 months are also excluded.

### Participant recruitment

**Figure 1:**
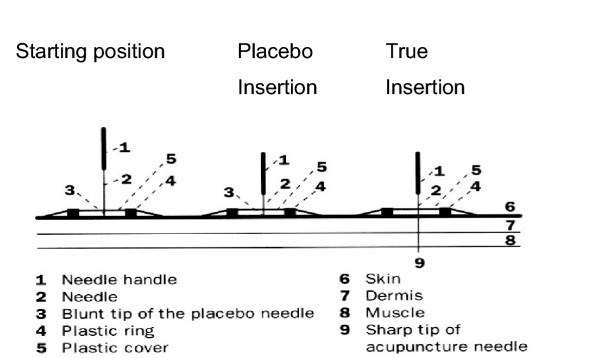
Placebo needle insertion

Eligible patients referred by their GP to the physiotherapy departments will be invited to take part. Recruitment will take place over 18 months and will operate in one of two ways (See Figure 1):

#### 1) Trial nurse

To identify potentially eligible patients, a trial nurse will review GP referral letters received by participating physiotherapy departments that fall within a feasibly commuteable geographical area of the Research Centre.

#### 2) Local physiotherapy assessor

A minimum of two members of the physiotherapy team will be involved at centres that fall outside the trial nurse's geographical area, one will be the nominated local assessor and a second will treat participants.

The nominated local physiotherapy assessor or trial nurse will perform an initial screen of the referrals to the physiotherapy department for potential participants. Potentially eligible patients will be posted information about the study, and their GP will be notified that the patient has been approached to take part. The GP will be asked to notify the Physiotherapy Department if they feel the patient is ineligible or unsuitable.

### Consent

A minimum of 48 hours after receiving the information leaflet, patients will be telephoned by the nominated local assessor/trial nurse (within 10 working days) to further screen eligibility. Information will be recorded on a standard proforma. For those patients either not eligible or not willing to be recruited, the proforma will be used to detail the reason for ineligibility, or reason for decline. Where patients are willing, two additional questions are asked to those that decline to participate in the trial to capture their treatment preference and expectations with respect to acupuncture and an advice & exercise treatment package. This information will be anonymised.

For patients willing to be recruited to the trial, an appointment is arranged for a research assessment at the patient's local physiotherapy department. After gaining verbal consent, the patient will be posted the baseline questionnaire to complete prior to their research appointment. At the research assessment visit the local physiotherapy assessor/trial nurse will perform a more detailed eligibility screen, explain the study, gain informed written consent to randomisation and conduct a baseline research interview and examination. Following consent to the study, the participant will be registered with the Research Centre by fax and allocated a unique trial number. The baseline assessment will be carried out blind to subsequent treatment allocation. An appointment will be made for the treating physiotherapist to begin treatment within 10 working days of the research assessment. Consent to treatment will be gained from each treating physiotherapist prior to commencing treatment, as is current physiotherapy practice. All participants will have an initial clinical physiotherapy assessment and treatment session of up to 40 minutes duration. During this session the physiotherapist will identify and record potential acupuncture points to be used should the participant be randomised to receive acupuncture (true or sham). A minimum of 6 and maximum of 10 points will be selected, based upon the participant's presentation and the clinical opinion of the physiotherapist. This will be carried out as part of the overall physical examination of the knee – the therapist will not draw the participant's attention to the localisation of acupuncture points to avoid raising their expectations about the possibility of receiving acupuncture. The advice and exercise package will then be started during this initial treatment visit.

### Randomisation

Randomisation will take place after this initial physiotherapy session. The treating physiotherapist will telephone the Research Centre at Keele University, during normal working hours. This methodology ensures that the initial physiotherapy assessment and advice and exercise package provided is performed blind to subsequent treatment allocation. The specific trial interventions will commence during the participant's second treatment visit. During the randomisation telephone call the physiotherapist will be asked to identify the selected acupuncture sites to check that the participant has received their first pre-randomisation treatment session. The physiotherapists will also be asked questions about their own expectations of the individual participant's likely clinical outcome and their beliefs about which treatment they would like the participant to receive. Participants recruited to the trial will then be randomised to one of the three trial interventions in a 1:1:1 ratio based on their unique trial number. Computerised third-party randomisation will be performed using random permuted blocks of 12 (blocked by treatment centre).

### Interventions

The interventions will be delivered within 10 working days of randomisation by experienced physiotherapists, trained in acupuncture to at least the minimum standard for basic membership of the Acupuncture Association of Chartered Physiotherapists (AACP) (35 hrs of training). The participant's GP will be contacted at the time of randomisation and asked to avoid co-interventions for the period of the trial wherever possible, but especially until the 6-week follow up has been completed. However, if the GP feels that symptoms are sufficiently troublesome to need further treatment this will be at the GPs discretion. Information about co-interventions will be collected in the follow-up questionnaires and by review of a sample of participants' clinical records.

#### a) Advice and exercise

Advice will be supplemented by a leaflet based on the **arc **Knee OA publication. This leaflet contains standard advice on the use of analgesia. If already using non-steroidal anti-inflammatory drugs, participants will be permitted to continue their stable dose. Participants will have the opportunity to discuss elements of the advice leaflet and exercise programme with their physiotherapist. In line with current practice, a maximum of 6 × 30-minute treatment sessions will be given over a period of 6 weeks. The advice and exercise programme has been developed through the use of reviews of current best evidence [23,27,55], clinical guidelines [9], a survey of current physiotherapy practice for knee pain [56], consensus workshop, and local physiotherapy practice.

The exercise programme will include concentric, eccentric, isometric and balance exercises. Specificity of training, particularly in the first 6 weeks, is important and therapists will aim for a mix of functional exercises, open & closed kinetic chain exercises and accelerated walking elements. They will also clearly identify a home exercise programme with set targets. The intensity of the exercises will be progressively increased at each session. Previously sedentary individuals who are relatively untrained will initially be prescribed exercise of a low intensity (eg. 1–3 sets of 8–12 repetitions of an exercise, 2–3 times per week). Progression to medium and high intensity exercise will occur only once adaptation to the current level of training has occurred.

Exercises will be prescribed and individualised for each participant by the treating physiotherapist from "Physio Tools" , a frequently used software package in physiotherapy. Hydrotherapy, group-based work, electrotherapy, additional acupuncture outside of the protocol and intra-articular injections will not be permitted. Participants may receive advice on the use of walking aids, and hot and cold applications. The key messages within the advice to participants include the common nature of knee problems and that rest for more than a day or two usually does more harm than good.

#### b) Advice and exercise plus true acupuncture

In addition to advice and exercise as detailed above, participants randomised to this group will receive 6 × 30-minute treatments of acupuncture, delivered over a period of 3 weeks. The acupuncture protocol is based on the concept of "treatment adequacy" which has been introduced by Ezzo et al [46] and Melchart et al [57] and has been shown recently to affect long term clinical outcome [58]. Physiotherapists delivering the treatment are provided with a choice of a total of 16 most commonly cited local and distal points from which they are required to chose between 6 – 10 points for each session. Local points available include: Sp 9, Sp 10, St 34, St 35, St 36, Xiyan, Gb 34 and trigger points. Distal points available include: LI 4, TH 5, Sp 6, Liv 3, St 44, Ki 3, BI 60 and Gb 41.

Treatment will be performed with sterilised disposable steel needles, 30 × 0.3 mm. The depth of the needle insertion should be between 0.5 – 2.5 cm depending on the points selected for treatment and the needles will be manipulated until de-qi sensation is achieved. Therapists allow 25 (min) to 35 (max) minutes between insertion of the last needle and cessation of treatment and during that time they are to revisit the needles as appropriate. If the sensation is maintained, they should manipulate each needle lightly, if the de-qi sensation is no longer there, they use stronger manipulation in order to elicit it.

Participants will be informed that they may or may not experience an aching, warm or `tingling' sensation from this type of stimulation. Therapists question participants at each session to ask them to describe the sensation they feel on needling, which is then recorded on a standard proforma. This also collects information on attendance, failed appointments, physiotherapist's diagnosis and whether any additional treatment modalities or specialist referrals are made.

#### c) Advice and exercise plus placebo acupuncture

In addition to advice and exercise as detailed above, participants randomised to this group will receive sham acupuncture which involves the placement of mock needles [47] upon a pre-defined set of points. The mock needle is a new device, which participants find indistinguishable from true acupuncture and has been used with success in a randomised trial comparing the effects of true versus placebo acupuncture in the treatment of shoulder pain [59]. The mock needle operates by allowing the shaft of the needle to collapse in the handle, creating an illusion of insertion (Figure 1 – adapted from [47])

The points chosen for the sham intervention receive no stimulation and participants will be told, as for the true acupuncture group, that they may or may not experience any particular sensation from this type of stimulation. The same parameters as for true acupuncture apply: placement of a minimum of 6 needles, and 6 × 30 minutes sessions within 3 weeks, with monitoring of elicited sensations.

### Audit of interventions

Using a standard proforma, the physiotherapists record the number and duration of treatment sessions each participant receives, plus details about the advice and exercises prescribed, the location and number of acupuncture points (where applicable) and any adverse reactions. The sensation that needling (true or placebo) evokes has been shown to be a significant correlate of acupuncture-induced analgesia (this has been a finding from both clinical and experimental studies). Hence, the sensation evoked from each treatment in the acupuncture groups will also be recorded. Acceptability and credibility of the interventions will be evaluated using a telephone follow-up at 2 weeks from the beginning of treatment and a questionnaire administered at 6 weeks [60].

### Baseline measures

Participants who give verbal consent to participate in the trial are posted a baseline questionnaire which they are asked to complete and bring with them to the research interview and examination. Information collected on this questionnaire is detailed in Table 2. These variables will be used to describe the study sample and as baseline measures of outcomes. Immediately following written informed consent, participants undergo a research interview and examination which follows a previously published schedule [66].

### Follow-up

Outcome measures will be performed at 2 weeks (Table 3), 6 weeks, 6 months and 12 months (Table 4). Follow-up assessments will be performed using a telephone call at 2 weeks after the first treatment and self-completed postal questionnaires at all other time points. Non-responders will be telephoned 2 weeks after mailing the follow-up questionnaire on up to 2 occasions and posted a replacement questionnaire with a reminder letter if there is still no response at 4 weeks.

### Sample size calculation

The primary outcome measure for this trial is the Western Ontario and McMaster Universities Osteoarthritis Index (WOMAC) pain sub-scale [61]. We have defined overall success as a 20% difference in the WOMAC pain sub-scale between true acupuncture and advice and exercise alone at 6 months. For this comparison, a minimum of 90 participants is needed in each group to reject the null hypothesis with 80% power and at a 5% significance level (two-tailed) [68]. As our trial will compare sham acupuncture with advice and exercise alone we have three groups and so need 270 participants. Allowing for a 30% drop out rate in those recruited to the trial, the total number of participants required to be randomised is 350.

### Analysis

Collection of data and statistical analysis will be performed blinded to treatment allocation. Analysis will be performed on an intention to treat basis and the primary outcome will also be analysed on a "per protocol basis".

Univariate analysis will be performed using t-tests to analyse numerical data and chi-square tests for categorical data. The clinical and demographic data collected at baseline will be inspected and if there are any important differences between the trial groups, these factors will be used as covariates. These analyses will be performed using ANOVA and logistic regression as appropriate.

The analysis of the secondary outcomes will be exploratory. A univariate analysis with respect to the different treatment will be performed for the WOMAC pain sub-scale at 6-weeks and 12-months and for the WOMAC functioning sub-scale at 6-weeks, 6- and 12-months. The global outcome assessment with be analysed both as an ordinal and a dichotomous variable (categories 1–3 defined as "success"). Moreover, area under the curve slopes will be calculated for each treatment group over the whole treatment period and compared.

Statistical significance will be set at the 5% level (two-tailed). Statistical analysis will be performed using Stata 7.0. The trial will be monitored by an independent Data Monitoring and Ethics Committee. No interim analysis of the primary or secondary outcomes will be undertaken during the trial period.

## Conclusions

The APEX trial is a major trial of physiotherapy treatment for knee pain. Obtaining participation by physiotherapists, across the regions of the West Midlands and Cheshire in 21 NHS Trusts, to work to agreed treatment protocols has been an important achievement. We have presented the rationale, design, and strategy for implementation of a multi-centre RCT examining whether acupuncture is a useful adjunct to usual physiotherapy care of advice and exercise for treating knee pain in older adults. The primary objective of the trial is to compare the clinical outcomes of true acupuncture plus advice and exercise, with advice and exercise alone for treating people aged 50 years and over referred directly from primary care with knee pain. The secondary objectives are to compare, at 6 weeks and 12 months, the clinical outcomes of adding true acupuncture to advice and exercise alone; to compare, at 6 weeks, 6 and 12 months, the clinical outcomes of placebo acupuncture plus advice and exercise, with advice and exercise alone; and to measure patients and therapists beliefs, preferences and expectations about the treatments being tested and to evaluate the association between these variables with clinical outcomes. The results of this trial will be presented as soon as they are available.

## Competing interests (medicine)

The authors declare that they have no competing interests.

## Authors' contributions

All authors participated in the design of the trial and drafting the manuscript. All authors have read and approved the final manuscript.

## Pre-publication history

The pre-publication history for this paper can be accessed here:



## Figures and Tables

**Figure 2:**
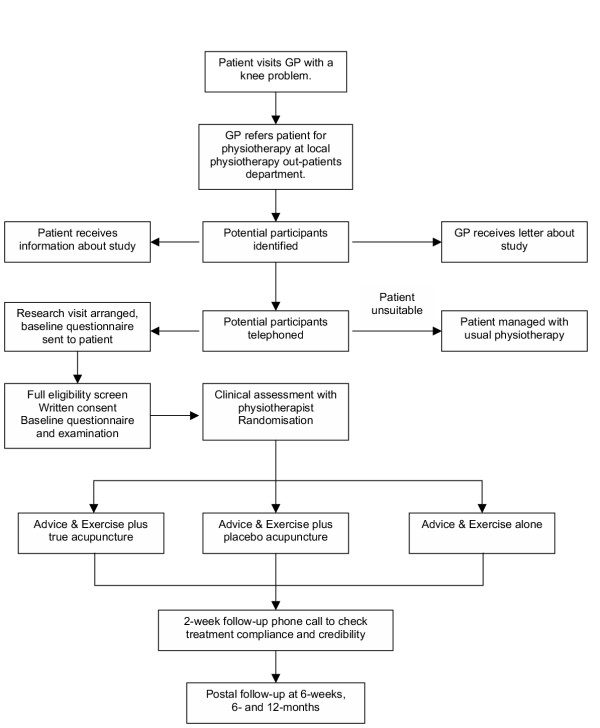
APEX Knee Study Schema

**Table 1:** Content of physiotherapist questionnaires

**Concept**	**Measurement method**	**Details**
*Information recorded on individual physiotherapists (collected before and after the trial)*		

Physiotherapist's information	- year qualified - gender - current clinical practice setting - clinical grade of current post - current work status - satisfaction with employment situation	community trust, acute trust staff/junior, senior II, senior I, extended scope practitioner/clinical specialist, superintendent/manager full time, part time very satisfied, satisfied, neither, unsatisfied, very unsatisfied

Use of acupuncture	- currently use it - length of time practising - use it for knee pain - member of AACP - previous training in acupuncture	<1 year, 1–3 years, 3–5 years, 5+ years no, associate, basic, full, advanced

Beliefs about knee pain (section B)	adapted from [53]	13 items on 7 point Likert scale: completely disagree to completely agree

Causes of knee problems	Illness Perceptions Questionnaire Revised (IPQ(R)) [54]	1 dimension: causes

Beliefs and expectations about treatment	- most helpful treatments for managing chronic knee pain - most effective - expected general improvement with each of the trial treatment - expect improvement in pain, movement and function with each specific trial treatment	OTC medication, prescribed medication, advice, exercise, acupuncture, rest advice and exercise, acupuncture, sham acupuncture 11 point numerical rating scale (NRS) 4 point scale: of great help, of some help, of little help, of no help

*Information recorded on individual patients (collected prior to randomisation)*		

Therapists perception of patients' knee severity		5 point Likert scale

Therapists prediction of likely outcome of patient's knee problem		5 point Likert scale

Therapists treatment preference for the patient		advice and exercise, acupuncture, no preference

Therapists expectation of treatment benefit for patient	- expectation of degree of improvement for the patient with each of the available treatments - expectations for improvement in specific outcomes of pain, function, and movement	11 point NRS/ 4 point Likert scale 11 point NRS/ 4 point Likert scale

**Table 2:** Content of baseline measures from self-completed questionnaire

**Concept**	**Measurement method**	**Details**
Bodily pain	- self-completed manikin	"In the past 4 weeks have you had pain that has lasted for one day or longer in any part of your body?"

Complaint specific functioning	Western Ontario and McMaster Universities OA index (WOMAC LK3.0) [61]	pain (0–20), stiffness (0–8), physical function (0–68) subscales

Participant-nominated principal functional problem		"Because of your knee, what one thing gives you the most problems?" 11 point NRS

Knee pain intensity and unpleasantness		11 point NRS

Illness perceptions	Illness Perceptions Questionnaire Revised (IPQ(R)) [54]	9 dimensions: illness coherence, treatment control, personal control, timeline (acute/chronic), timeline (cyclical), consequences, emotional representation, identify, causes

Patient's self-efficacy	Arthritis Self-Efficacy Scale [62]	11 items on 10 point NRS

Experiences and preferences for treatment	- previous experience with exercise - preference for trial treatments - perceived helpfulness of trial treatments - outcome expectancy with trial treatments	yes, no 5 point Likert scale 4 point Likert scale 11 point NRS

Quality of life	EuroQol EQ-5D [63]	Summary score and 100 mm VAS score

Occupational characteristics	- current employment status - satisfaction with employment situation - current/recent job title - socio-economic classification - work loss in last 6 months due to knee problem	working full time, working part time, working in the home, unemployed/seeking employment, not working due to ill health/disability, student, retired very satisfied, satisfied, neither, unsatisfied, very unsatisfied SOC 2000 [64,65]

Demographic characteristics	- date of birth, gender - marital status	single, married, widowed, divorced, cohabiting, single

Anthropometric characteristics	-self-reported height -self-reported weight	

**Table 3:** Content of 2-week phone call measures

**Concept**	**Measurement method**	**Details**
Treatment compliance	- number of times visited trial physiotherapist so far - extent of compliance with prescribed exercises	5 point Likert scale

Change in knee symptoms	- any change - specific change	yes, no 5 point Likert scale

Knee pain intensity		11 point numerical rating scale (NRS)

Credibility of interventions	- confidence in received treatment - logic of received treatment Adapted from [60]	5 point Likert scale

Experiences and expectations	- treatment met expectations - expected improvement with treatment	5 point Likert scale 11 point NRS

Treatment preference	- want to change treatment receiving	yes, no, not sure

**Table 4:** Content of follow-up measures from self-completed questionnaire collected at 6-weeks, 6- and 12-months

**Concept**	**Measurement method**	**Details**
Bodily pain	- self-completed manikin	"In the past 4 weeks have you had pain that has lasted for one day or longer in any part of your body?"

Complaint specific functioning	Western Ontario and McMaster Universities OA index (WOMAC LK3.0) [61]	pain (0–20), stiffness (0–8), physical function (0–68) subscales

Participant-nominated principal functional problem		"Because of your knee, what one thing gives you the most problems?" 11 point NRS

Knee pain intensity and unpleasantness		11 point NRS

Bothersomeness of knee problem		5 point scale: not at all, slightly, moderately, very much, extremely

Illness perceptions	Illness Perceptions Questionnaire Revised (IPQ(R)) [54]	9 dimensions: illness coherence, treatment control, personal control, timeline (acute/chronic), timeline (cyclical), consequences, emotional representation, identify, causes

Patient's self-efficacy	Arthritis Self-Efficacy Scale [62]	11 items measuring patient's "certainty"

Global outcome	[67]	6 point scale: completely recovered, much improved, improved, same, worse, much worse

Credibility of interventions	- confidence in received treatment - logic of received treatment Adapted from [58]	5 point Likert scale

Side effects	- self-reported side effects	nausea/vomiting, drowsiness/sleepiness, bruising, fainting, headaches, soreness to joints

Health care utilisation for knee problem	- consultations	GP, district nurse, physiotherapist, hospital consultant, osteopath, chiropractor, homeopath, acupuncturist

Medication for knee problem	- prescribed/OTC medication	medicine name, dose, number taken

Experiences and preferences for treatment	- previous experience with exercise - preference for trial treatments - perceived helpfulness of trial treatments - outcome expectancy with trial treatments	yes, no 5 point Likert scale 4 point Likert scale 11 point NRS

Quality of life	EuroQol EQ-5D [63]	Summary score and VAS score

Occupational characteristics	- current employment status - satisfaction with employment situation - current/recent job title - socio-economic classification - work loss since last questionnaire due to knee problem	working full time, working part time, working in the home, unemployed/seeking employment, not working due to ill health/disability, student, retired very satisfied, satisfied, neither, unsatisfied, very unsatisfied SOC 2000 [63,64]

Demographic characteristics	- date of birth, gender - marital status	single, married, widowed, divorced, cohabiting, single

## References

[B1] McAlindon TE, Cooper C, Kirwan JR, Dieppe PA (1992). Knee pain and disability in the community. Br J Rheumatol.

[B2] O'Reilly SC, Muir KR, Doherty M (1996). Screening for pain in knee osteoarthritis: which question?. Ann Rheum Dis.

[B3] Scott DL, Shipley M, Dawson A, Edwards S, Symmons DP, Woolf AD (1998). The clinical management of rheumatoid arthritis and osteoarthritis: strategies for improving clinical effectiveness. Br J Rheumatol.

[B4] Hawley DJ, Wolfe F (1991). Pain, disability, and pain/disability relationships in seven rheumatic disorders: a study of 1,522 patients. J Rheumatol.

[B5] Creamer P, Flores R, Hochberg MC (1998). Management of osteoarthritis in older adults. Clin Geriatr Med.

[B6] Hochberg MC, Altman RD, Brandt KD, Clark BM, Dieppe PA, Griffin MR, Moskowitz RW, Schnitzer TJ (1995). Guidelines for the medical management of osteoarthritis. Part II. Osteoarthritis of the knee. Arthritis Rheum.

[B7] Lane NE, Thompson JM (1997). Management of osteoarthritis in the primary-care setting: an evidence-based approach to treatment. Am J Med.

[B8] Altman RD, Lozada CJ (1998). Practice guidelines in the management of osteoarthritis. Osteoarthritis Cartilage.

[B9] The Primary Care Rheumatology Society (1999). The management of osteoarthritis – Guidelines.

[B10] Mazzuca SA, Brandt KD, Katz BP, Dittus RS, Freund DA, Lubitz R, Hawker G, Eckert G (1997). Comparison of general internists, family physicians, and rheumatologists managing patients with symptoms of osteoarthritis of the knee. Arthritis Care Res.

[B11] Coyte PC, Hawker G, Croxford R, Attard C, Wright JG (1996). Variation in rheumatologists' and family physicians' perceptions of the indications for and outcomes of knee replacement surgery. J Rheumatol.

[B12] Arthritis Care Report (2004). Osteoarthritis Nation: the most comprehensive UK report of people with osteoarthritis.

[B13] Pencharz JN, Grigoriadis E, Jansz GF, Bombardier C (2002). A critical appraisal of clinical practice guideline for the treatment of lower-limb osteoarthritis. Arthritis Res.

[B14] Wainapel SF, Thomas AD, Kahan BS (1998). Use of alternative therapies by rehabilitation outpatients. Arch Phys Med Rehabil.

[B15] Eisenberg DM, Kessler RC, Foster C, Norlock FE, Calkins DR, Delbanco TL (1993). Unconventional medicine in the United States. Prevalence, costs and patterns of use. N Engl J Med.

[B16] Eisenberg DM, Davis RB, Ettner SL, Appel S, Wilkey S, Van Rompay M, Kessler RC (1998). Trends in alternative medicine in the United States: 1990–1997. Results of a follow-up national survey. JAMA.

[B17] Thomas K, Fall M, Parry G, Nichol J (1995). National survey of access to complementary health care via general practice.

[B18] Filshie J, White AR (1998). Medical acupuncture.

[B19] Ernst E, White A (2000). The BBC survey of complementary medicine use in the UK. Complement Ther Med.

[B20] Vickers A (2000). Recent advances: Complementary medicine. BMJ.

[B21] Woollam CHM, Jackson AO (1998). Acupuncture in the management of chronic pain. Anaesthesia.

[B22] Zollman C, Vickers A (1999). ABC of complementary medicine: Users and practitioners of complementary medicine. BMJ.

[B23] Felson DT, Lawrence RC, Hochberg MC, McAlindon T, Dieppe PA, Minor MA, Blair SN, Berman BM, Fries JF, Weinberger M, Lorig KR, Jacobs JJ, Goldberg V (2000). Osteoarthritis: new insights. Part 2: Treatment approaches. Ann Intern Med.

[B24] Hurley M, Dziedzic K, Bearne L, Sim J, Bury T (2001). Clinical and cost effectiveness of physiotherapy in the management of older people with common rheumatological conditions.

[B25] Puett DW, Griffin MR (1994). Published trials of nonmedicinal and noninvasive therapies for hip and knee osteoarthritis. Ann Intern Med.

[B26] Foley A, Halbert J, Hewitt T, Crotty M (2003). Does hydrotherapy improve strength and physical function in patients with osteoarthritis – a randomised controlled trial comparing a gym-based and a hydrotherapy based strengthening programme. Ann Rheum Dis.

[B27] Van Baar ME, Assendelft WJJ, Dekker J, Oostendorp RAB, Bijlsma JWJ (1999). Effectiveness of exercise therapy in patients with osteoarthritis of the hip or knee. Arthritis Rheum.

[B28] Evcik D, Sonel B (2002). Effectiveness of a home-based exercise therapy and walking program on osteoarthritis of the knee. Rheumatol Int.

[B29] Rejeski WJ, Focht BC, Messier SP, Morgan T, Pahor M, Penninx B (2002). Obese, older adults with knee osteoarthritis: weight loss, exercise and quality of life. Health Psychol.

[B30] Thomas KS, Muir KR, Doherty M, Jones AC, O'Reilly SC, Bassey EJ (2002). Home based exercise programme for knee pain and knee osteoarthritis: randomised controlled trial. BMJ.

[B31] Topp R, Woolley S, Hornyak J, Khuder S, Kahaleh B (2002). The effect of dynamic versus isometric resistance training on pain and functioning among adults with osteoarthritis of the knee. Arch Phys Med Rehabil.

[B32] Lewek MD, Rudolph KS, Snyder-Mackler L (2004). Quadriceps femoris muscle weakness and activation failure in patients with symptomatic knee osteoarthritis. J Orthop Res.

[B33] Jordan KM, Arden NK, Doherty M, Bannwarth B, Bijlsma JW, Dieppe P, Gunther K, Hauselmann H, Herrero-Beaumont G, Kaklamanis P, Lohmander S, Leeb B, Lequesne M, Mazieres B, Martin-Mola E, Pavelka K, Pendleton A, Punzi L, Serni U, Swoboda B, Verbruggen G, Zimmerman-Gorska I, Dougados M, Standing Committee for International Clinical Studies Including Therapeutic Trials ESCISIT (2003). EULAR Recommendations 2003: an evidence based approach to the management of knee osteoarthritis: Report of a Task Force of the Standing Committee for International Clinical Studies including therapeutic trials (ESCISIT). Ann Rheum Dis.

[B34] Han JS, Pomeranz B, Stux G (1989). Central neurotransmitters and acupuncture analgesia. Scientific Bases of Acupuncture.

[B35] Andersson S, Lundeberg T (1995). Acupuncture – from empiricism to science: Functional background to acupuncture effects in pain and disease. Med Hypotheses.

[B36] Haker E, Egekvist H, Bjerring P (2000). Effect of sensory stimulation (acupuncture) on sympathetic and parasympathetic activities in healthy subjects. J Autonom Nerv Syst.

[B37] Knardahl S, Elam M, Olausson B, Wallin BG (1998). Sympathetic nerve activity after acupuncture in humans. Pain.

[B38] Ter Riet G, Kleijnen J, Knipschild P (1990). Acupuncture and chronic pain: A criteria-based meta-analysis. J Clin Epidemiol.

[B39] Smith LA, Oldman AD, McQuay HJ, Morre RA (2000). Teasing apart quality and validity in systematic reviews: an example from acupuncture trials in chronic neck and back pain. Pain.

[B40] Ernst E, White AR (1997). A review of problems in clinical acupuncture research. Am J Chin Med.

[B41] Takeda W, Wessel J (1994). Acupuncture for the treatment of pain of osteoarthritic knees. Arthritis Care Res.

[B42] Christensen BV, Iuhl IU, Vilbek H, Bullow H-H, Dreijer NC, Rasmussen HF (1992). Acupuncture treatment of severe knee osteoarthrosis: a long term study. Acta Anaesthesiol Scand.

[B43] Anonymous (1998). NIH Consensus Conference: Acupuncture. JAMA.

[B44] Deluze C, Bosia L, Zirbs A, Chantraine A, Vischer TL (1992). Electroacupuncture in fibromyalgia: Results of a controlled trial. BMJ.

[B45] Berman BM, Swyers JP (1999). Complementary medicine treatments for fibromyalgia syndrome. Baillieres Best Pract Res Clin Rheumatol.

[B46] Ezzo J, Hadhazy V, Birch S, Lao L, Kaplan G, Hochberg M, Berman B (2001). Acupuncture for osteoarthritis of the knee: a systematic review. Arthritis Rheum.

[B47] Streitberger K, Kleinhenz J (1998). Introducing a placebo needle into acupuncture research. Lancet.

[B48] White P, Lewith G, Hopwood V, Prescott P (2003). The placebo needle, is it a valid and convincing placebo for use in acupuncture trials? A randomised, single-blind, cross-over pilot trial. Pain.

[B49] Brinkhaus B, Becker-Witt C, Jena S, Linde K, Streng A, Wagenpfeil S, Irnich D, Hummelsberger J, Melchart D, Willich SN (2003). Acupuncture Randomized Trials (ART) in patients with chronic low back pain and osteoarthritis of the knee – design and protocols. Forsch Komplementarmed Klass Naturheilkd.

[B50] Tukmachi E, Jubb R, Dempsey E, Jones P (2004). The effect of acupuncture on the symptoms of knee osteoarthritis-an open randomised controlled study. Acupunct Med.

[B51] Ernst E (2004). The placebo needle, is it a valid and convincing placebo for use in acupuncture trials? A randomised, single-blind, cross-over pilot trial [letter]. Pain.

[B52] NHS Executive (1997). R&D in primary care National Working Group report (the Mant report).

[B53] Rainville J, Bagnall D, Phalen L (1995). Health care providers' attitudes and beliefs about functional impairments and chronic back pain. Clin J Pain.

[B54] Moss-Morris R, Weinman J, Petrie K, Horne R, Cameron LD, Buick D (2002). The Revised Illness Perception Questionnaire (IPQ-R). Psychol Health.

[B55] Hurley MV (1999). The role of muscle weakness in the pathogenesis of osteoarthritis. Rheum Dis Clin North Am.

[B56] Foster NE, Barlas P, Dziedzic K, Daniels J, Gray R (2000). Current physiotherapy management of knee osteoarthritis informs a clinical trial [abstract]. Rheumatology.

[B57] Melchart D, Linde K, Fischer P, Berman B, White A, Vickers A, Allais G (2004). Acupuncture for idiopathic headache (Cochrane Review). In The Cochrane Library.

[B58] He D, Bo Veiersted K, Hostmark AT, Ingulf Medbo J (2004). Effect of acupuncture treatment on chronic neck and shoulder pain in sedentary female workers: a 6-month and 3-year follow-up study. Pain.

[B59] Kleinhenz J, Streitberger K, Windeler J, Güßbacher A, Mavridis G, Martin E (1999). Randomised clinical trial comparing the effects of acupuncture and a newly designed placebo needle in rotator cuff tendinitis. Pain.

[B60] Vincent CA, Lewith G (1995). Placebo controls for acupuncture studies. J R Soc Med.

[B61] Bellamy N (1996). WOMAC Osteoarthritis Index. A User's Guide.

[B62] Lorig K, Chastain RL, Ung E, Shoor S, Holman HR (1989). Development and evaluation of a scale to measure perceived self-efficacy in people with arthritis. Arthritis Rheum.

[B63] Kind P, Dolan P, Gudex C, Williams A (1998). Variations in population health status: results from a United Kingdom national questionnaire survey. BMJ.

[B64] Office for National Statistics (2000). Standard occupational classification 2000 The coding index London.

[B65] Office for National Statistics (2002). The National Statistics Socio-economic classification user manual Version 1 London.

[B66] Peat G, Thomas E, Handy J, Wood L, Dziedzic K, Myers H, Wilkie R, Duncan R, Hay E, Hill J, Croft P (2004). The Knee Clinical Assessment Study – CAS(K): Prospective study of knee pain and knee osteoarthritis in the general population. BMC Musculoskeletal Disorders.

[B67] van der Windt DA, Koes BW, Deville W, Boeke AJ, de Jong BA, Bouter LM (1998). Effectiveness of corticosteroid injections versus physiotherapy for treatment of painful stiff shoulder in primary care: randomised trial. BMJ.

[B68] Norman GR, Streiner DL (1994). Biostatistics: the bare essentials.

